# Comparison of clinical efficacy of methylprednisolone and serratiopeptidase for reduction of postoperative sequelae after lower third molar surgery

**DOI:** 10.4317/jced.51868

**Published:** 2015-04-01

**Authors:** Mouneshkumar Chappi D., Kandagal V. Suresh, Manisha R. Patil, Rajendra Desai, David P. Tauro, Shiva Bharani K.N.S., Mushtaq I. Parkar, Harsha V. Babaji

**Affiliations:** 1 MDS, Senior lecturer, Department of Oral & maxillofacial surgery, School of Dental Sciences, Krishna Institute of Medical Sciences, Deemed University, Karad, Satara (District), Maharashtra (State), India; 2 MDS, Senior lecturer, Department of Oral Medicine and Radiology, School of Dental Sciences, Krishna Institute of Medical Sciences, Deemed University, Karad, Satara (District), Maharashtra (State), India; 3 DNB, Senior Resident, Department of Pediatrics, RCSM Government Medical College, Kolhapur (District), Maharashtra (State), India; 4 MDS, Professor, Department of Oral & maxillofacial surgery, College of dental sciences, Davangere (District), Karnataka (State), India; 5 MDS, Professor & Head, Department of Oral & maxillofacial surgery, College of dental sciences, Davangere (District), Karnataka (State), India; 6 MDS, Professor, Department of Oral & maxillofacial surgery, College of Dental Sciences, Davangere (District), Karnataka (State), India; 7 MDS, Professor, Department of Oral & maxillofacial surgery, School of Dental Sciences, Krishna Institute of Medical Sciences, Deemed University, Karad, Satara (District), Maharashtra (State), India; 8 Senior lecturer, Department of Oral & maxillofacial surgery, College of Dental Sciences, Davangere (District), Karnataka (State), India

## Abstract

**Background:**

Surgical removal of mandibular third molars results in some degree of post-operative pain, swelling and trismus. These can be controlled by proper administration of local anesthesia, careful bone removal, minimal trauma to adjacent soft tissues and administration of methylprednisolone and serratiopeptidase drugs. The aim of the present study was to compare the efficacy of methylprednisolone and serratiopeptidase in controlling post-operative pain, swelling and trismus after surgical removal of impacted mandibular third molars.

**Material and Methods:**

The subjects were divided into two groups of 50 patients each undergoing surgical removal of mandibular third molars. Group A was given methylprednisolone 4mg orally every 8th hourly and Group B was given serratiopeptidase 10 mg every 12th hourly orally. Post-operatively pain, swelling and trismus were evaluated at the end of 1st, 3rd and 5thday.

**Results:**

The results of this study showed that methylprednisolone is an effective analgesic, while serratiopeptidase has moderate analgesic activity. Serratiopeptidase is more effective than methylprednisolone in controlling post surgical swelling and trismus. Hence combination of these two drugs would be very effective than individual drug when widespread post-operative sequelae are expected after surgical removal of impacted lower third molars.

**Conclusions:**

We conclude that methylprednisolone affords better pain relief while serratiopeptidase exerts better anti-inflammatory and anti-swelling effects in the post-operative period. Synergistic combinations of these two drugs would however prove to be more effective when extensive post-operative sequelae are expected.

** Key words:**Methylprednisolone, serratiopeptidase, pain, swelling, trismus, third molar.

## Introduction

Surgical removal of teeth in oral surgery inevitably requires bone removal, which sometimes results in some degree of post-operative pain, swelling and trismus. Minimal trauma to adjacent soft tissues and proper wound closure minimizes pain, swelling and trismus. These can be controlled by proper administration of local anesthesia, good flap reflection, careful bone removal and minimal trauma to adjacent soft tissues with appropriate wound closure techniques (1). Pharmacologic strategies for minimizing the clinical manifestations of surgical injury are therefore, logically directed at blocking the formation or inhibiting the effects of mediators of acute inflammation. Corticosteroids are routinely employed to control the sequelae of inflammation in third molar surgeries (2). The effectiveness of glucocorticoids as anti-inflammatory agents was first reported by Hench and Kendallin 1949, using cortisone to treat rheumatoid arthritis. Steroids act by suppression of leukocyte and macrophage accumulation at the inflammatory site by interfering with capillary dilatation, fibrin deposition and prevention of prostaglandin synthesis by inhibiting the arachidonic acid cascade (1-4).

In a study conducted by Elhag *et al.* for establishing the anti-inflammatory effect of methylprednisolone, the author found that the methylprednisolone group had mean swelling volumes significantly lower than that of the control (5). A study conducted by Akhan has suggested that, the pre-operative use of methylprednisolone appears to be a safe and effective method of reducing post-operative complications in third molar surgery (6). Al-Khateeb and Nusair on the use of serratiopeptidase for reduction of post operative pain, swelling and trismus after third molar surgery, concluded that significant reduction in swelling could be achieved with the use of serratiopeptidase (7).

Since very few researchers studied the efficacy of methylprednisolone and serratiopeptidase in postoperative management pain, swelling and trismus; very limited information was available in the literature regarding these two drugs and so far no comparative studies are available on methylprednisolone and serratiopeptidase in reduction of postoperative sequelae after third molar surgery. Hence an attempt has been made to study the efficacy of these two drugs in patients undergoing surgical extraction of third molar.

## Material and Methods

Present study was carried out on 100 patients on an out-patient basis in the department of oral and maxillofacial surgery. Patients were divided randomly into two groups, irrespective of age and sex - each group consisting of 50 patients.

Inclusion criteria included ASA Grade-1 patients and absence of pathologic changes teeth and surrounding tissues.

-Exclusion criteria includes;

• Patients with genetically transmitted bleeding disorder

• Extraoral and intraoral swelling with cellulitis in the area of surgery

• History of systemic illness 

• Patient on other drugs such as NSAIDS and Steroids 

A written consent was obtained from all the pateints after being explained about the study purpose and the nature of the surgical procedure performed and drugs prescribed after it. Ethical clearance was obtained from the institutional ethical committee. Pre-operative investigations which included a complete hemogram and serology in all the subjects. Patients who satisfied the inclusion and exclusion criteria were taken up for 3rd molar extraction under local anesthesia. Methylprednisolone 4mg orally 8th hourly for 5 day was post operatively prescribed to patients in Group-A while Group-B was prescribed with serratiopepti-dase 10mg orally 12th hourly for 5 days. The patients were recalled for a follow – up visit on 1st, 3rd and 5th day post-operatively.

Pre-operative facial measurements were obtained by marking 5 points on the face i.e. at the symphysis, mastoid, angleof mandible, lateral canthus of eye and the ala of nose. Measurements were taken from a point on the angle of mandible which served as a common point, to the tragus, lateral canthus of eye, ala of the nose, angle of the mouth and soft-tissue pogonion. Using a black silk suture measurements were recorded in millimeters.

-Surgical procedure for extraction of impacted mandibular third molar:

Under routine aseptic precautions, local anesthesia was secured with 2% lignocaine hydrochloride with 1: 80,000 adrenaline, using the conventional inferior alveolar nerve block. A standard Terrance-Ward incision was placed. A mucoperiosteal flap was raised exposing the underlying bone. Bone guttering was carried out buccally and on the distal aspect of the impacted tooth using a round bur. Odontectomy was performed whenever indicated to facilitate tooth removal. The tooth was removed from the socket with the help of dental elevators. The socket was irrigated with povidine iodine and saline after the sharp bony edges were smoothened. Complete hemostasis was achieved and wound closed was obtained using 3-0 silk suture. The follow up was carried out on the 1st, 3rd and 5th post-operative day. All the patients were under standard antibiotic coverage with cap. amoxicillin 500 mg thrice daily for 5 days postoperatively.

The post-operative swelling, cheek girth and mouth opening were measured in the same manner as was done preoperatively. Pain assessment was made using subjective visual analog scale. Mouth opening was measured between the incisal edges of the maxillary and mandibular central incisors. The data were statistically analyzed and interpreted by using un paired t – test and Chi-square test.

## Results

The present study was aimed at evaluating the individual efficacies and comparing methylprednisolone 4mg orally and serratio-peptidase 10 mg orally in controlling post-operative pain, swelling and trismus after surgical removal of impacted mandibular third molars.

- Comparison of swelling size between two groups 

**Figure 1:**
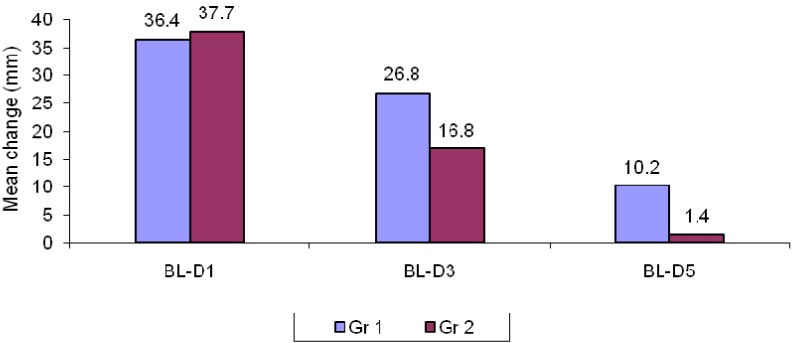
Comparison of changes in swelling measurements in two groups.

The mean size of the swelling in group I was 36.4 ± 27.6, 26.8 ± 38.4 and 10.2 ± 28.3 on day 1, 3 and 5 respectively whereas in group II the mean size was 37.7 ± 24.9, 16.8 ± 18.5 and 1.4 ± 8.2 on day 1, 3 and 5 respectively. When compared, the results were statistically insignificant between group I and group II on the day 1 and 3 (*p*=0.80 and 0.10 respectively). However, statistical significance was observed on day 5. (Un paired t–test). Thus, serratiopeptidase has better efficacy in controlling post-operative edema than methylprednisolone ( Table 1, Fig. 1).

**Table 1:**
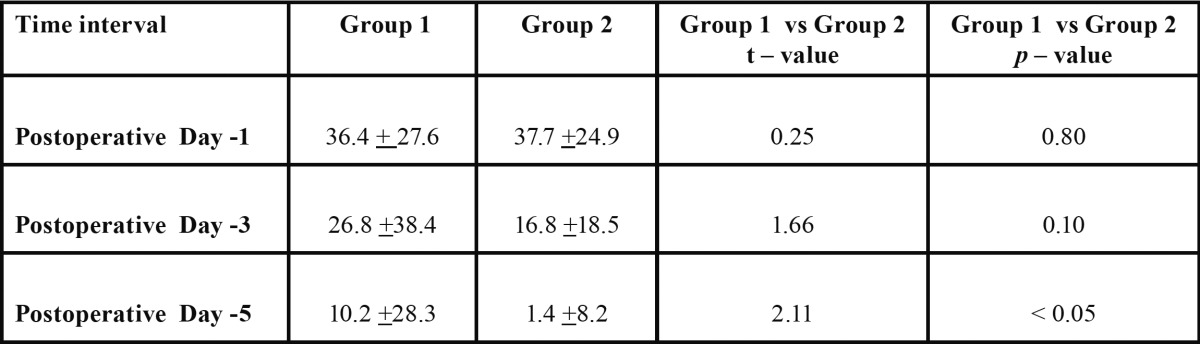
Comparison of post operative changes in swelling size between two groups.

- Comparison of pain between two groups on different days 

**Figure 2:**
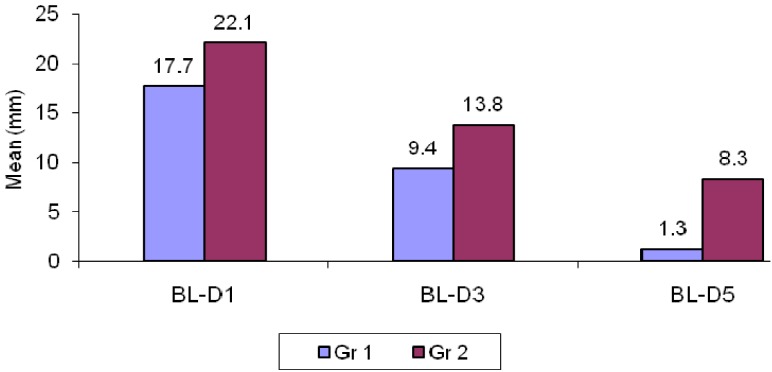
Comparison of postoperative pain in two groups.

Under the mild pain category, group I had 5 patients reporting mild pain on day 1, 6 patients on day 3 and 22 patients on day 5, whereas in group II, 1 patient reported mild pain on day 1 but no pain was reported on day 3 (n=0) and 5 (n=0). Under the moderate pain category, group I had 9, 39 and 28 patients on day 1, 3 and 5 respectively, whereas group II had 1, 14 and 50 patients on day 1, 3 and 5 respectively. Under the severe pain category, group I had 36 patients on day 1 and 5 patients on day 3 but no pain was reported on day 5 (n=0), whereas group II had 48 and 36 patients on day 1 and 3 respectively. No pain was reported on day 5 (n=0). These observations indicated statistically significant differences between the two groups (*p*<0.05), suggesting that pain control efficacy in group I was better than in group II (Chi-square test) ( Table 2, Fig. 2).

**Table 2:**
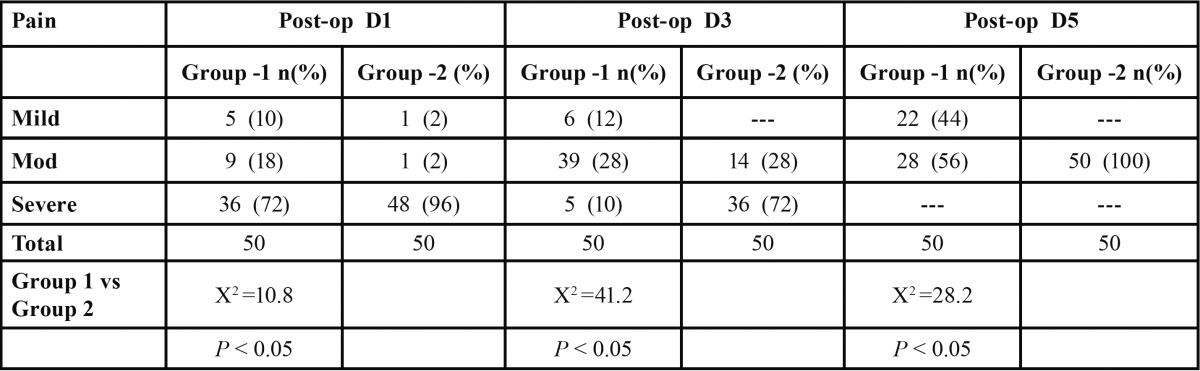
Comparison of postoperative pain between two groups on different days.

- Comparison of changes in trismus between two groups 

The mean difference in mouth opening post-operatively on day 1 in group I was 17.7 ± 5.5mm; this difference reduced to 9.4 ± 4.0 and 1.3 ± 2.8 on day 3 and 5 respectively. The mean difference in mouth opening post-operatively on day 1 in group II was 22.1 ± 4.2mm; this difference reduced to 13.8 ± 5.6 and 8.3 ± 4.6 on day 3 and 5 respectively.

**Figure 3:**
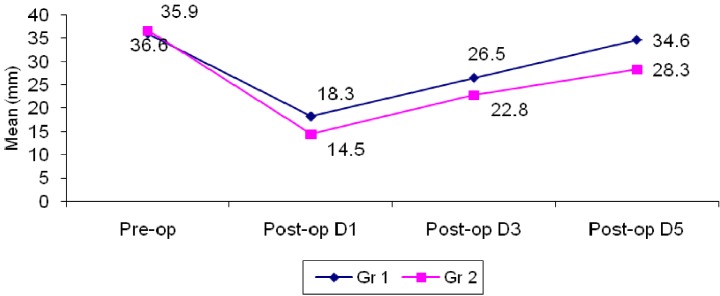
Comparison of trismus measurement in two groups.

When the results were compared, statistical significance was observed between group I and II (*P*<0.001) i.e. resolution of trismus was better in group II than in group I. (Un paired t–test) This attests to the probable fact that serratiopeptidase is better than methylprednisolone in resolving trismus post-operatively ( Table 3, Fig. 3).

**Table 3:**
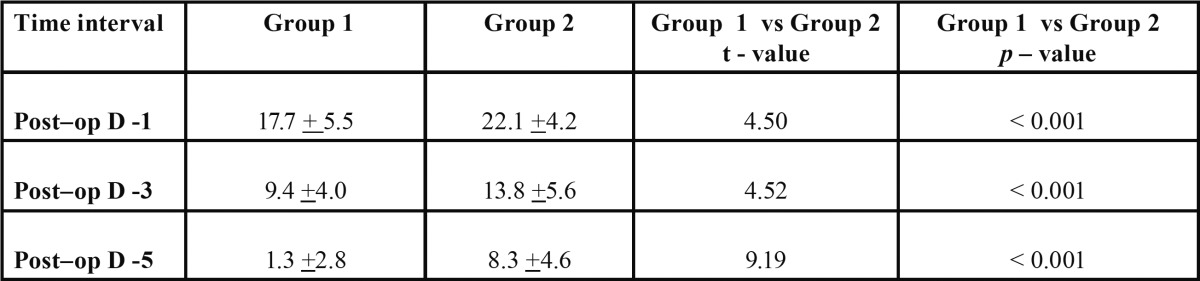
Comparison of changes in trismus between two groups.

## Discussion

Surgical removal of impacted 3rd molar is usually perceived as a procedure causing considerable pain and swelling to the patient. Good surgical technique and gentle handling of tissues will minimize these symptoms. The body’s defense mechanism to cell injury is inflammation. When tissue damage occurs, large quantities of histamine, bradykinin, serotonin and other substances are released into the surrounding area (8,9). These substances, especially histamine causes local vasodilatation, thereby increasing blood flow to the damaged area. The permeability of venous capillaries and venules is also increased. This inflammatory process is necessary for healing to occur, but often excessive inflammation causes the patient unnecessary pain, swelling and trismus (10). Glucocorticosteroids are the group of steroids that possess anti-inflammatory properties secreted by the adrenal cortex (11). One of the most important actions of corticosteroids is the suppression of inflammation by interfering with capillary dilatation, edema formation, fibrin deposition, leucocytes migration and phagocytosis (12,13).

Enzymes are considered as extremely potent substances and the possibility of their therapeutic application is attractive. Enzymes are derived from bacteria (streptokinase and streptodornase), from plant (papase and bromelain) and from animals (trypsin and chymotrypsin). Serratiopeptidase is a proteolytic enzyme produced by a microorganism Serratia species. This endopeptidase is prepared from the culture broth of genus Serratio SP E-15 (one of the enteric bacilli in silk worm). It has high enzyme activity, including a potent anti-inflammatory action, anti-swelling as well as bradykinin-decomposing activity and it also enhances the antibiotic action at the site of the lesion (4,7).

Troullos *et al.* suggested that intravenous administration of methylprednisolone combined with low oral doses in effective management of postoperative surgical sequale. In our study, methylprednisolone 4 mg was administered orally for 5 days, which showed apparently good improvement clinically (14).

Pederson concluded that steroid administration is effective in reducing post-operative complaints when pronounced post-operative reaction can be expected and that short-term administration is safe in the absence of contraindications for such administration. In the present study, methylprednisolone was found to be safe in the absence of contraindications (15).

Panwar SK. concluded that a single preoperative dose of prednisolone reduces postoperative swelling. Statistical analysis and observations were done to determine the difference in swelling, trismus and pain among the control group and study group. A single pre-operative dose of oral prednisolone helped the patient to cope the postoperative sequela, without causing major side effects. In our study, methylprednisolone 4 mg administered orally for 5 days, showed good satisfactory results (16).

Esch *et al.* used serratiopeptidase for reduction of postoperative swelling in upper ankle joint surgery and concluded that significant reduction in swelling had been achieved post- operatively with the use of serratiopeptidase. In the present study similar effects were observed with serratiopeptidase proving it to be better in reducing swelling than methylprednisolone (17).

Al-Khateeb and Nusair investigated the ability of serratiopeptidase to reduce post-operative pain, swelling and trismus after third molar surgery. There was a significant reduction in the pain intensity and extent of cheek swelling in the serratiopeptidase group at the 2nd, 3rd and 7th post-operative days. The present study also indicated similar results in which the serratiopeptidase group showed significantly reduced trismus and swelling on the day 1, 3 and 5 post-operatively. However, this enzyme preparation did not exert an appreciable analgesic effect post-operatively (7).

Murugesan K. found that dexamethasone was more effective in reducing swelling and pain when compared with serratiopeptidase, however both had same effect on trismus. In our study, serratiopeptidase 10 mg orally produced an appreciable anti-edema effect and had no apparent effect on rate of wound healing (18).

## Conclusions

In the present study two different drugs methylprednisolone and serratiopeptidase were compared for clinical efficacy on post operative pain, swelling and trismus after surgical removal of mandibular third molars. Methylprednisolone affords better pain relief while serratiopeptidase exerts better anti-inflammatory effects in the post-operative period. Synergistic combinations of these two drugs would however prove to be more effective when extensive post-operative sequele are expected, but randomized case-control research trials need to be conducted in the future to attest to this probable fact.
